# Radiation-Associated Lung Adenosquamous Carcinoma Diagnosed by Robotic-Assisted Bronchoscopy With Cone-Beam CT Guidance and Treated With Robotic Superior Segmentectomy: A Case Report

**DOI:** 10.7759/cureus.116213

**Published:** 2026-09-14

**Authors:** Beauty Etukudoh, Noah Gordon, Raisa Munshi, Mae Leef, Abdul Hamid Alraiyes

**Affiliations:** 1 Internal Medicine, Advocate Lutheran General Hospital, Park Ridge, USA; 2 Interventional Pulmonology, Advocate Lutheran General Hospital, Park Ridge, USA

**Keywords:** adenosquamous carcinoma, augmented fluoroscopy, cone-beam ct, pet-avid pulmonary nodule, post-radiation fibrosis, radial ebus, radiation-associated lung cancer, radiation-induced malignancy, robotic-assisted bronchoscopy, segmentectomy

## Abstract

Prior thoracic or chest-wall radiotherapy carries a small but meaningful risk of second primary malignancy after a prolonged latency period. Chronic post-radiation changes may also complicate the evaluation of new pulmonary lesions, particularly when fibrosis, traction bronchiectasis, and architectural distortion are present. Advances in robotic-assisted bronchoscopy combined with radial endobronchial ultrasound (rEBUS), augmented fluoroscopy, and cone-beam computed tomography (CBCT) may facilitate localization and tissue diagnosis of suspicious peripheral pulmonary lesions in this setting.

We report the case of an 82-year-old woman with a remote smoking history and recurrent myxofibrosarcoma of the left upper back. At Year 0, she underwent surgical resection followed by adjuvant radiotherapy to 60 Gy in 30 fractions. At Year 10, she developed a high-grade local recurrence that was managed with radical re-excision followed by electron re-irradiation to an additional 60 Gy in 30 fractions. Whole-body PET/CT performed during evaluation of the recurrence showed no evidence of distant metastatic disease. She did not receive systemic therapy for her myxofibrosarcoma.

During surveillance following re-irradiation, imaging identified an enlarging spiculated left lower lobe lesion with associated traction bronchiectasis adjacent to post-treatment changes in the posterior thoracic region. PET/CT demonstrated increased fluorodeoxyglucose (FDG) uptake within the lesion, with a maximum standardized uptake value (SUVmax) of 2.9, increasing suspicion for malignancy, although inflammatory processes may also demonstrate FDG uptake. Robotic-assisted bronchoscopy with rEBUS, CBCT, and augmented fluoroscopic guidance established the diagnosis of primary lung adenocarcinoma.

Following multidisciplinary evaluation, the patient underwent robotic-assisted superior segmentectomy with systematic lymph node dissection and additional margin resection. Final pathology demonstrated a 2.2 cm well-differentiated adenosquamous carcinoma confined to the lung parenchyma, with negative sampled lymph nodes and a negative final margin. Surveillance imaging approximately seven months after resection demonstrated no new pulmonary nodules or masses.

The temporal and anatomic relationship of the lesion to prior chest-wall radiotherapy raised consideration of a radiation-associated malignancy; however, causality cannot be established from a single case. Available 2024 boost-plan dosimetry demonstrated limited average pulmonary exposure, although cumulative dose at the exact lesion site was not available. This case illustrates the successful use of robotic bronchoscopy, rEBUS, CBCT, and augmented fluoroscopy for lesion localization and tissue diagnosis in complex post-treatment thoracic anatomy without establishing superiority over alternative diagnostic approaches.

## Introduction

Radiation-associated lung cancer is a rare but important potential long-term complication of thoracic and chest-wall radiotherapy [1-6]. Although modern radiation techniques have improved significantly over the years, adjacent lung parenchyma may still receive incidental radiation exposure, resulting in chronic fibrosis, traction bronchiectasis, architectural distortion, and, in rare cases, secondary malignancy after a prolonged latency period [1-8]. Distinguishing malignancy from benign post-radiation change can be particularly difficult because fibrotic lung tissue may obscure or closely mimic cancer on routine imaging [2,3,7].

Several mechanisms have been proposed to contribute to radiation-associated carcinogenesis, including persistent oxidative stress, endothelial injury, genomic instability, and impaired DNA repair [1-8]. These mechanisms remain incompletely established, and the development of a malignancy near a previously irradiated region does not independently establish a causal relationship.

With the development of advanced bronchoscopic technologies such as robotic-assisted bronchoscopy (RAB), radial endobronchial ultrasound (rEBUS), augmented fluoroscopy, and cone-beam computed tomography (CBCT), evaluation of peripheral pulmonary lesions has become more precise in selected patients [9]. These modalities may assist with lesion localization, confirmation of tool-in-lesion positioning, and targeted sampling, particularly in patients with distorted post-treatment anatomy. However, superiority over conventional diagnostic techniques cannot be inferred from a single case.

We present a case of pulmonary adenosquamous carcinoma arising adjacent to a previously irradiated posterior thoracic region nearly 11 years after the initial treatment of chest-wall myxofibrosarcoma. The lesion was diagnosed using RAB with rEBUS, CBCT, and augmented fluoroscopic guidance and was subsequently managed with robotic superior segmentectomy following multidisciplinary discussion.

## Case presentation

The patient was an 82-year-old woman with a remote smoking history and recurrent myxofibrosarcoma of the left upper back who was found to have a new pulmonary lesion during routine surveillance imaging. She was clinically well at the time of evaluation and denied fever, chills, new cough, dyspnea, or chest pain.

At Year 0, she was initially diagnosed with a 4 cm myxofibrosarcoma of the left upper back following excision of a presumed lipoma. The initial surgical margins were positive. Staging CT chest demonstrated a small right middle lobe perifissural nodule without mediastinal or hilar lymphadenopathy or pleural effusion. She subsequently underwent radical re-excision, which demonstrated residual myxofibrosarcoma measuring ≤1 cm involving the deep subcutaneous tissue and skeletal muscle. Final margins were negative but close, with tumor approximately 1.1 mm from the deep and superior margins. Given the risk of local recurrence, she received adjuvant radiotherapy to 60 Gy in 30 fractions.

At Year 10, she developed a new nodule near the inferior aspect of the prior surgical field. Pathology demonstrated recurrent high-grade myxofibrosarcoma (FNCLCC (Fédération Nationale des Centres de Lutte Contre Le Cancer) grade III) with undifferentiated/pleomorphic features. She underwent radical re-excision with bilateral paraspinous muscle flap reconstruction. Final pathology demonstrated a 4.2 cm recurrent tumor with negative but close margins, several measuring ≤3 mm. There was no lymphovascular invasion, tumor necrosis was approximately 5%, and the mitotic rate was 10 mitoses/mm².

Whole-body PET/CT performed during evaluation of the recurrent sarcoma demonstrated no evidence of active residual or distant metastatic disease, with only mild postsurgical uptake in the posterior chest wall. She did not receive systemic therapy or chemotherapy for her myxofibrosarcoma.

Given the high-grade recurrence and close surgical margins, she underwent adjuvant re-irradiation. The re-irradiation course used electron therapy, with 50 Gy delivered in 25 fractions followed by a 10-Gy boost in five fractions, for a total prescribed dose of 60 Gy. The boost used 9- and 12 MeV electron fields with a 0.5 cm bolus.

Review of the available boost-plan dose-volume histogram demonstrated a mean dose of 88.9 cGy and a maximum dose of 1062.5 cGy to the contoured lungs excluding gross target volume. These values represent the available boost plan only. A cumulative composite dose incorporating the initial and repeat radiation courses at the exact site of the subsequently diagnosed pulmonary tumor was not available.

On Day 0, surveillance CT chest demonstrated an enlarging 3-4 cm region of somewhat spiculated curvilinear and possibly fibrotic change in the superior left lower lobe with associated traction bronchiectasis and reticular density (Figures 1, 2). The abnormality was adjacent to post-treatment changes in the posterior thoracic region. Although the appearance could represent fibrotic change, malignancy could not be excluded.

**Figure 1:**
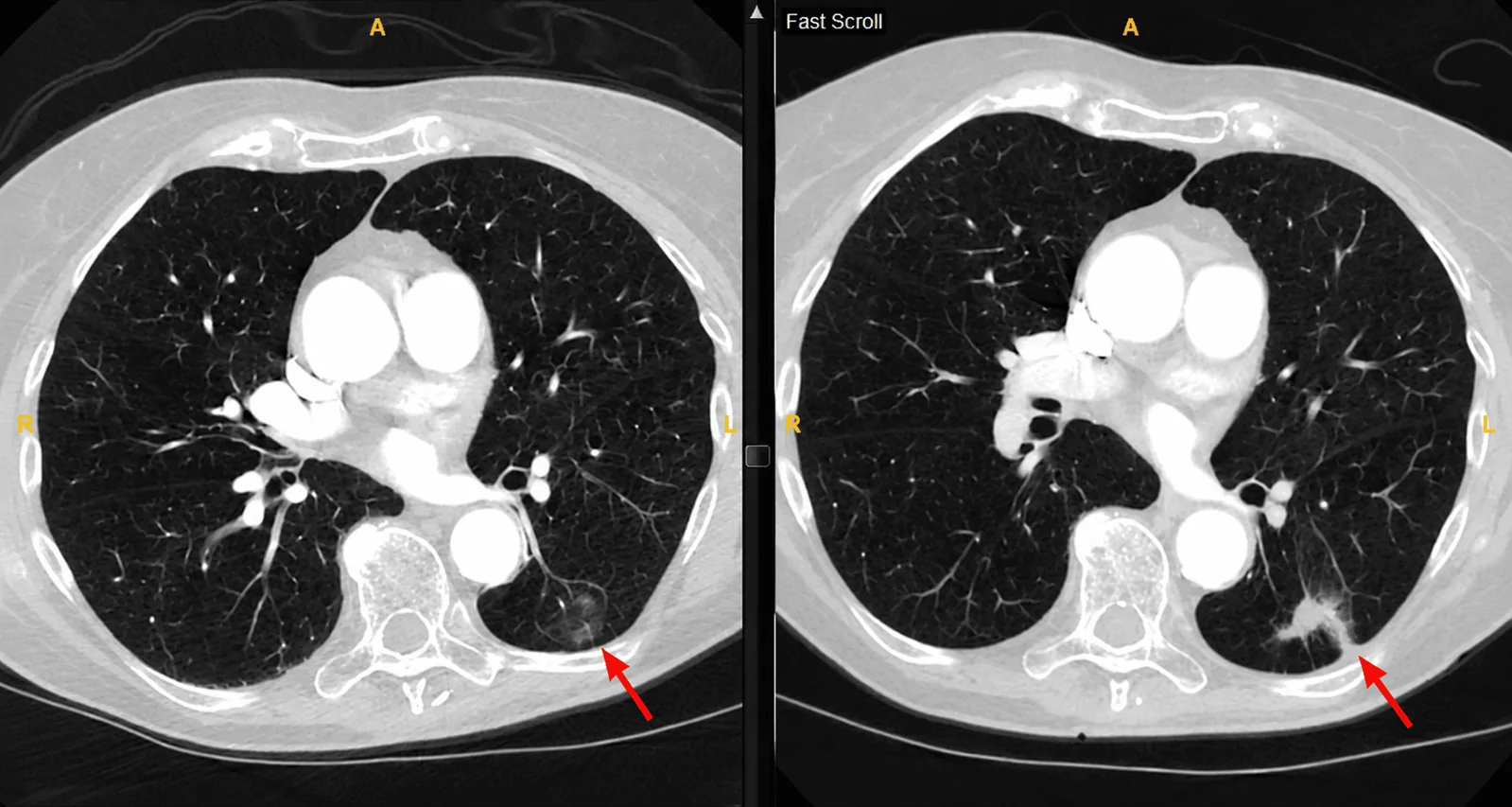
Development of new left lower lobe pulmonary lesion on surveillance CT imaging. Axial CT chest images demonstrating interval development of a spiculated left lower lobe pulmonary nodule adjacent to areas of post-radiation fibrosis and traction bronchiectasis within the previously irradiated thoracic field. Comparison imaging highlights progression from subtle parenchymal abnormality to a more discrete suspicious lesion.

**Figure 2:**
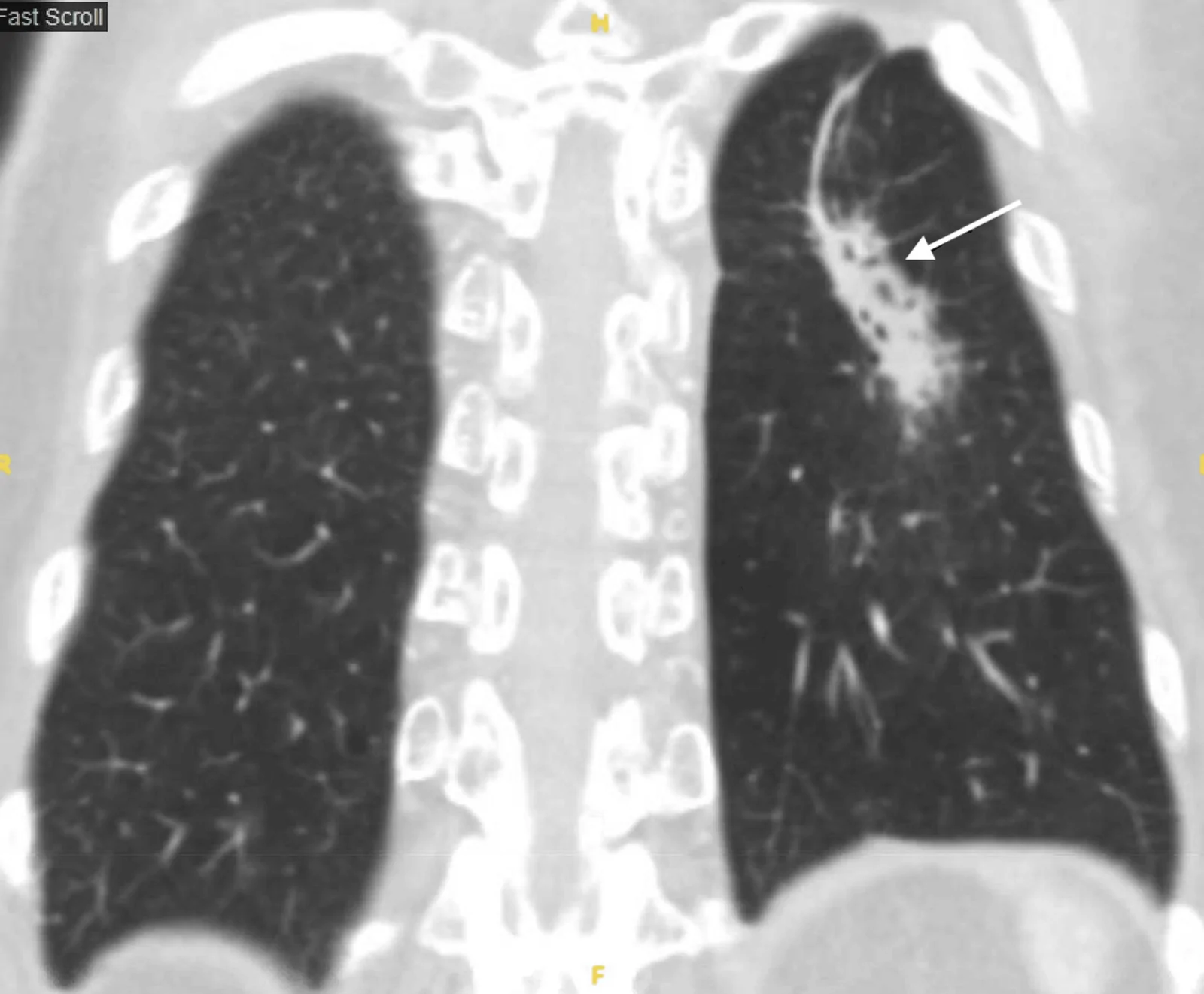
Coronal CT chest demonstrating post-radiation architectural distortion and left lower lobe lesion. Coronal CT imaging demonstrating fibrotic post-radiation changes involving the left posterior thoracic lung parenchyma with associated traction bronchiectasis and adjacent spiculated pulmonary lesion concerning for malignancy.

Subsequent PET/CT demonstrated a 1.9 cm spiculated left lower lobe nodule with increased fluorodeoxyglucose (FDG) uptake and a maximum standardized uptake value (SUVmax) of 2.9 (Figures 3, 4). In the setting of interval progression and suspicious morphology, this increased concern for malignancy, although FDG uptake is not specific for malignancy and may also occur with inflammatory processes. There was no hypermetabolic lymphadenopathy.

**Figure 3:**
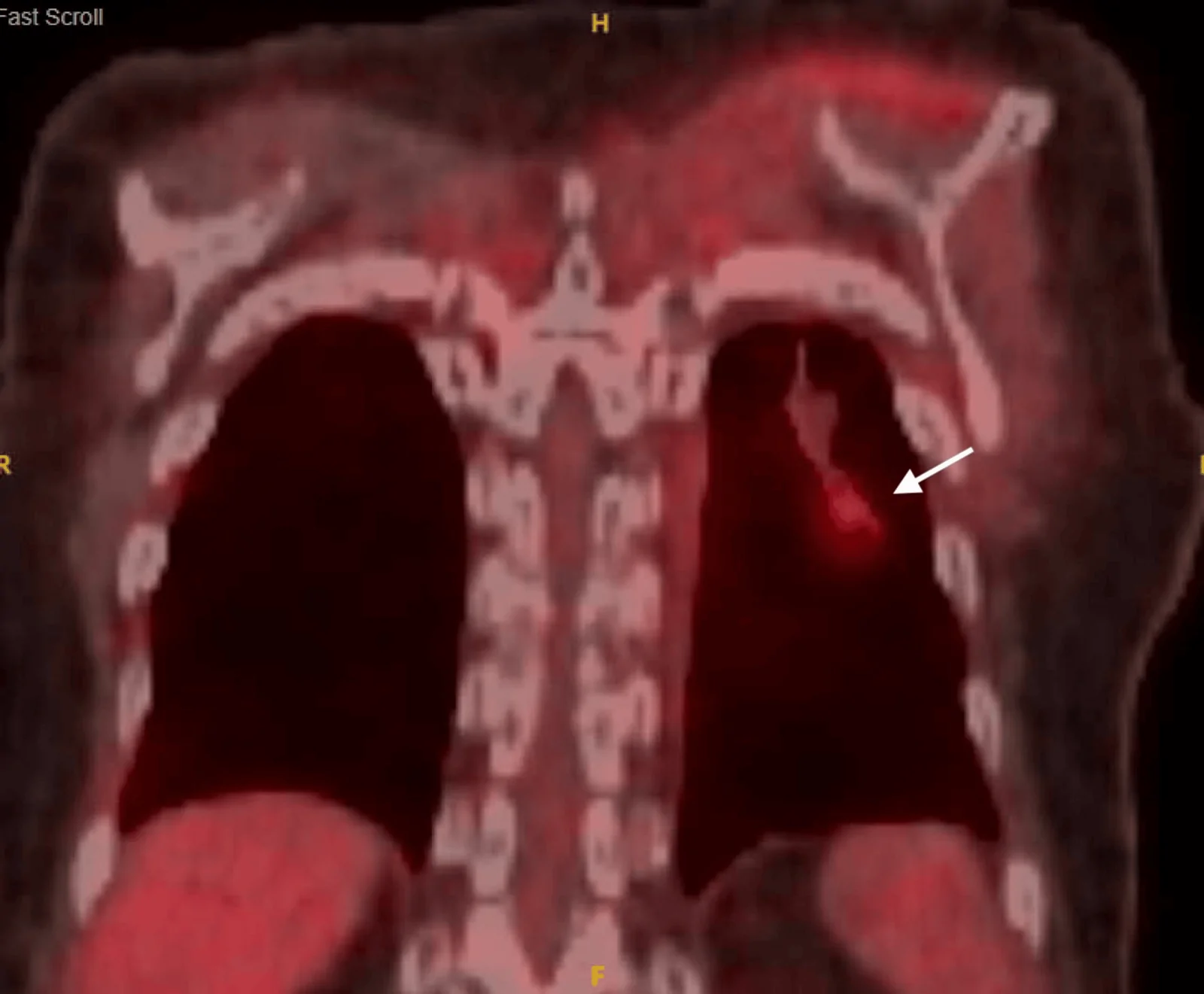
Coronal fused PET/CT demonstrating focal FDG avidity of the pulmonary lesion. Coronal fused PET/CT image demonstrating focal FDG uptake within the 1.9 cm spiculated left lower lobe pulmonary nodule (SUVmax 2.9), corresponding to the suspicious lesion identified on CT imaging. FDG, fluorodeoxyglucose; SUVmax, maximum standardized uptake value.

**Figure 4:**
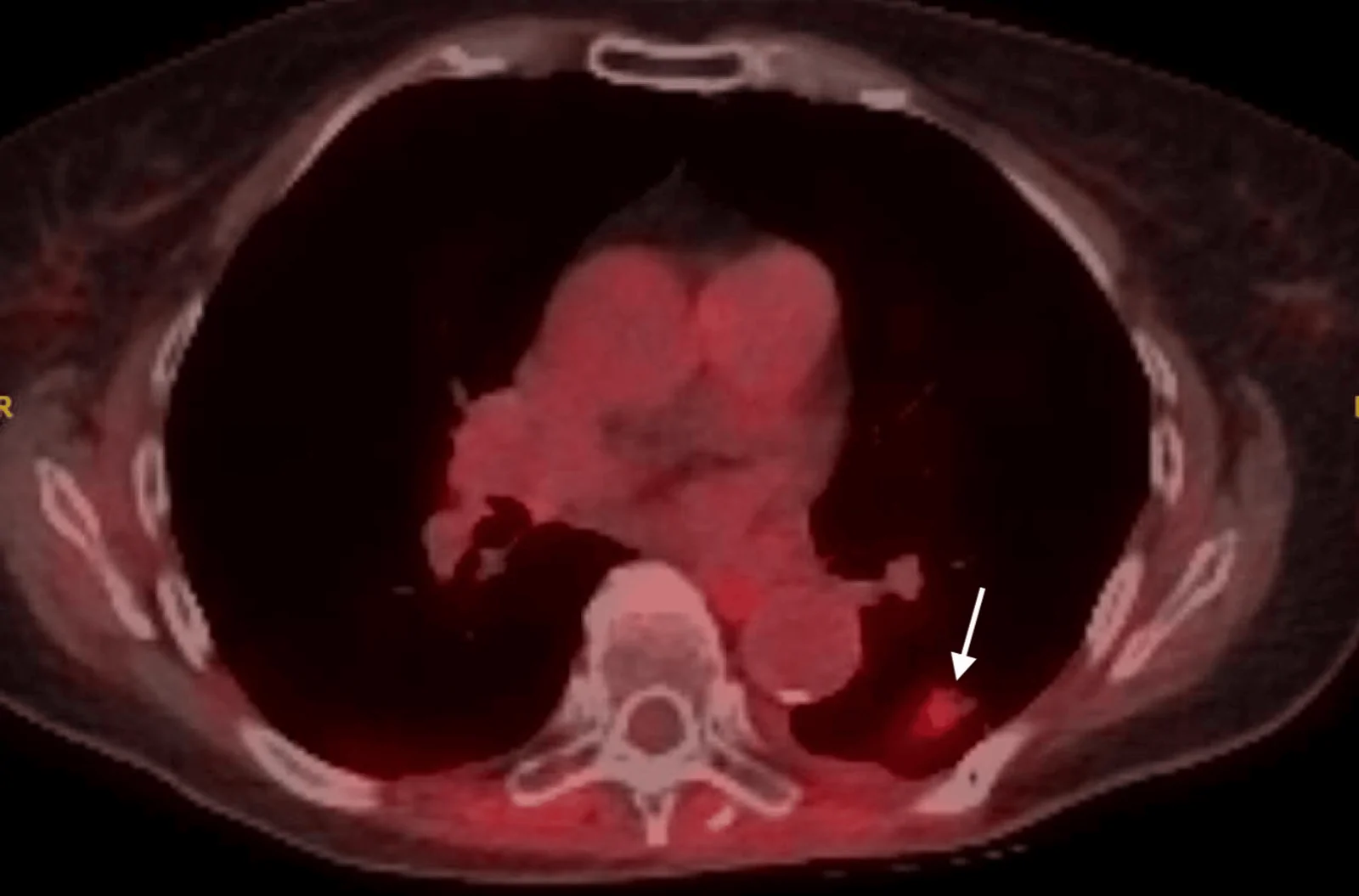
Axial fused PET/CT demonstrating metabolically active left lower lobe lesion. Axial fused PET/CT image demonstrating focal FDG uptake within the peripheral left lower lobe lesion corresponding to the suspicious pulmonary nodule identified on CT imaging. FDG, fluorodeoxyglucose.

At Week 5, the patient underwent RAB with rEBUS and CBCT guidance [9]. Procedural planning was focused on localization and sampling of the most suspicious component of the lesion. Given the posterior location of the lesion in the distal lung, the procedure was performed in the prone position following endotracheal intubation to reduce CT-to-body divergence and dependent posterior atelectasis, which could otherwise interfere with lesion localization [9].

Intraprocedural CBCT was used together with augmented fluoroscopic pathway planning to navigate to the posterior left lower lobe target (Figures 5, 6). The green circular overlays represent the augmented fluoroscopic targeting pathway used to maintain localization of the lesion during respiratory motion throughout inspiration and expiration, allowing continued focus on the target during navigation. Augmented fluoroscopic guidance and CBCT confirmation demonstrated tool-in-lesion positioning prior to tissue acquisition (Figure 7).

**Figure 5:**
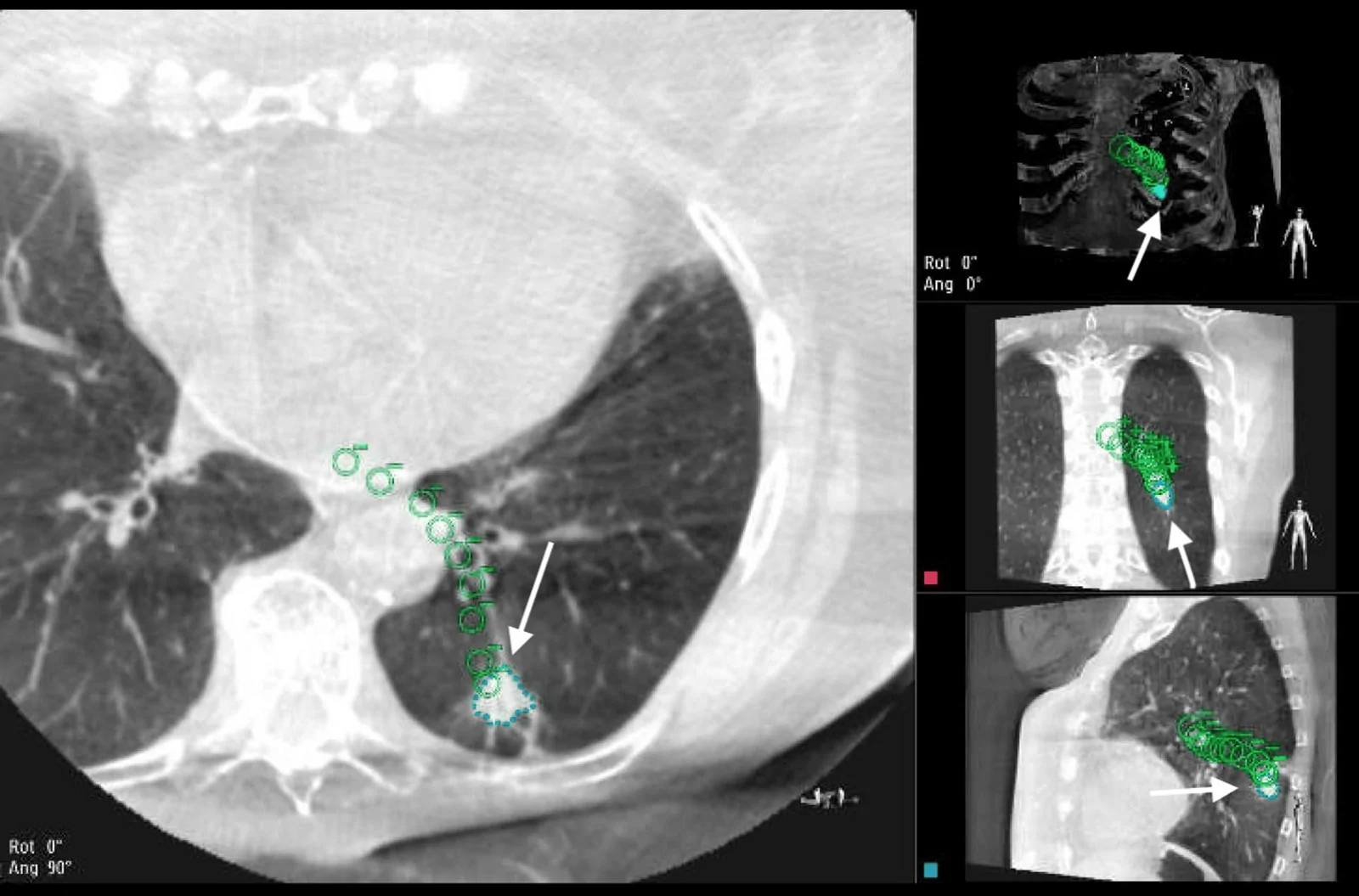
Cone-beam CT segmentation and augmented fluoroscopic planning during robotic-assisted bronchoscopy. Intraprocedural cone-beam CT images demonstrating augmented fluoroscopic pathway planning to the posterior left lower lobe target. The green circular overlays represent the augmented fluoroscopic targeting pathway used to maintain localization of the lesion throughout inspiration and expiration during navigation.

**Figure 6:**
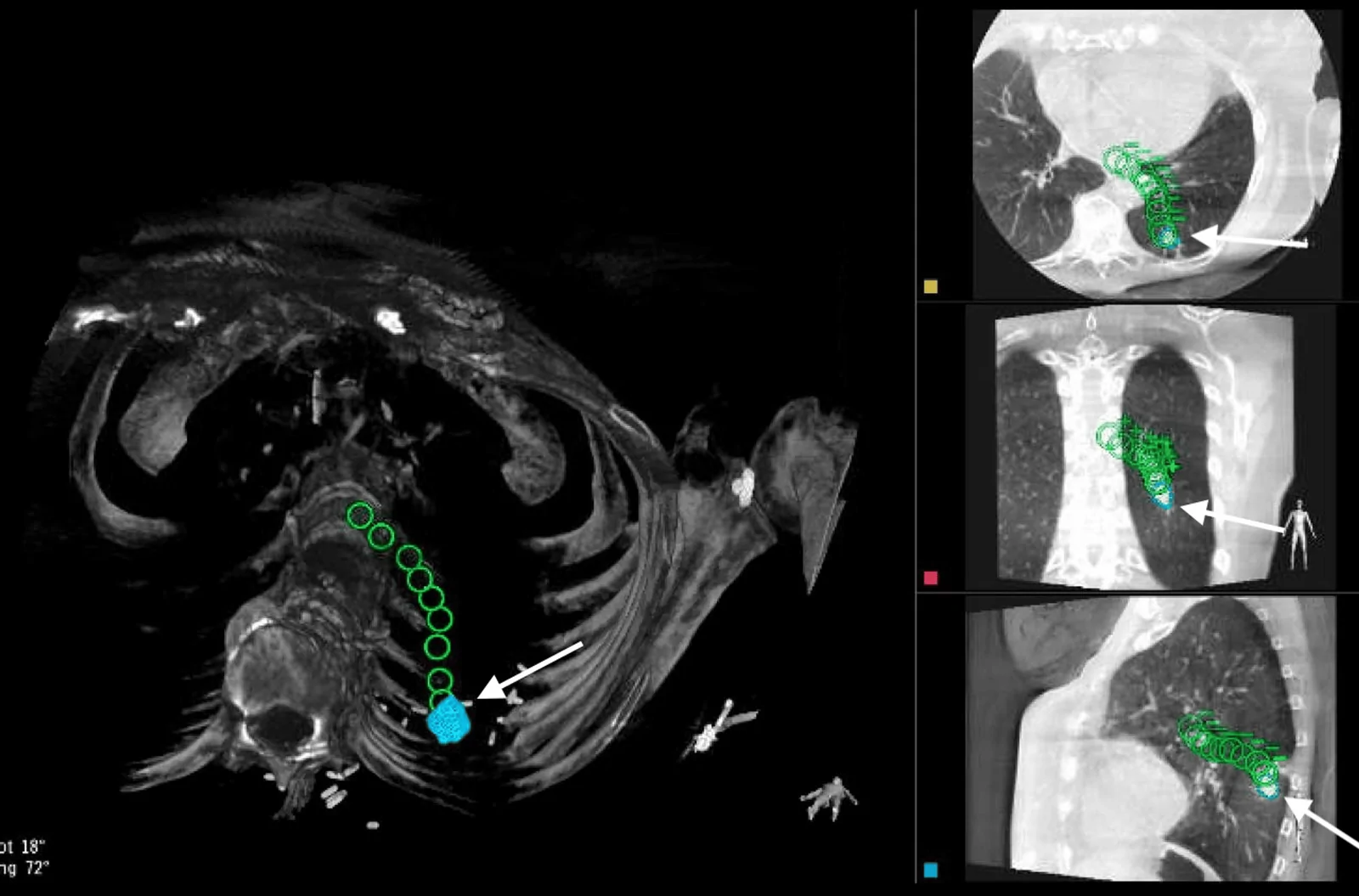
Three-dimensional CBCT reconstruction demonstrating robotic bronchoscopic navigation pathway. Three-dimensional CBCT reconstruction demonstrating the augmented fluoroscopic navigational pathway to the posterior left lower lobe target during robotic-assisted bronchoscopy. CBCT, cone-beam computed tomography.

**Figure 7:**
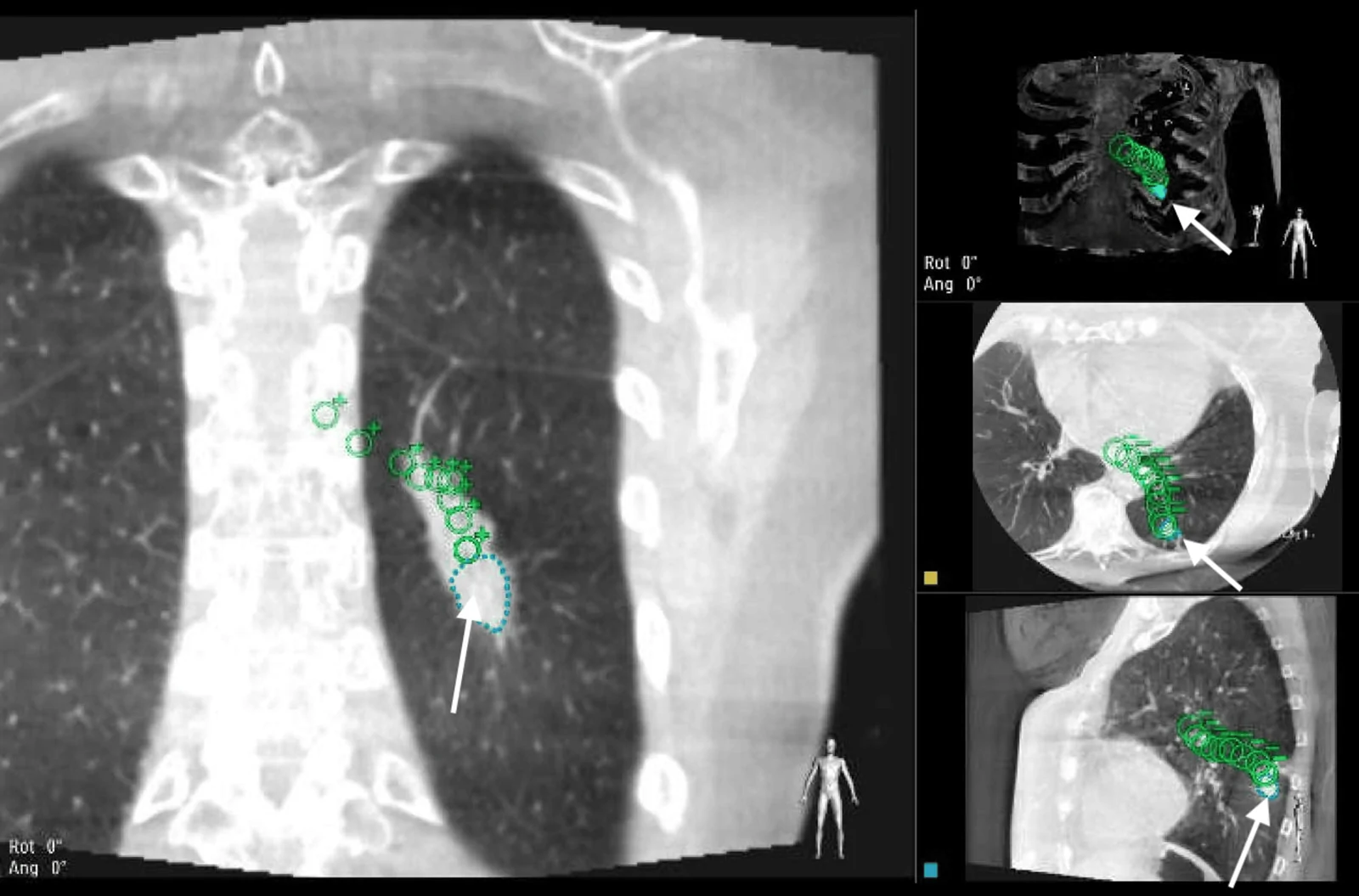
Coronal CBCT with augmented fluoroscopic tool-in-lesion confirmation. Coronal CBCT image demonstrating successful localization of the biopsy catheter within the segmented PET-avid lesion using augmented fluoroscopic guidance prior to tissue acquisition. CBCT, cone-beam computed tomography.

This multimodal approach facilitated targeted sampling of the intended region; however, the relative contribution of each technology and any comparative advantage over alternative biopsy approaches cannot be determined from this case.

Transbronchial cryobiopsy of the targeted lesion demonstrated pulmonary adenocarcinoma with an acinar growth pattern associated with fibroelastotic scar (Figure 8). Complete mediastinal and hilar staging was subsequently performed using EBUS-guided transbronchial needle aspiration, which demonstrated lymphoid tissue without evidence of malignancy. Detailed diagnostic immunohistochemical stains, including TTF-1, p40, and CK5/6, were not available in the records reviewed and, therefore, are not reported.

**Figure 8:**
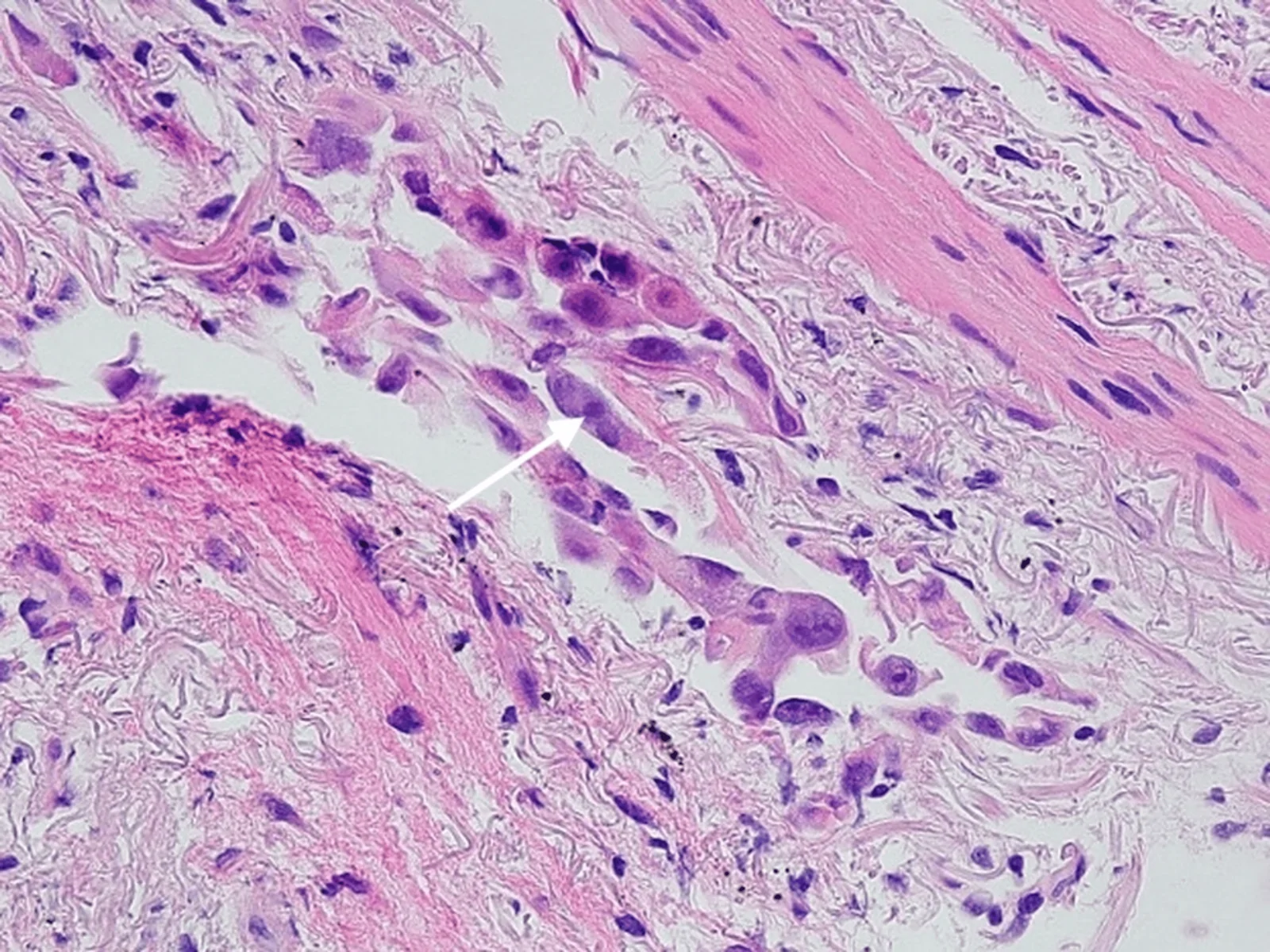
Histopathology of transbronchial cryobiopsy demonstrating pulmonary adenocarcinoma. H&E stain (×400) demonstrating malignant gland-forming epithelial cells infiltrating a background of fibroelastotic scar, consistent with pulmonary adenocarcinoma obtained by robotic-assisted transbronchial cryobiopsy. H&E, hematoxylin and eosin.

Preoperative pulmonary function testing demonstrated preserved spirometry and lung volumes, with forced expiratory volume in 1 second (FEV_1_) of 1.40 L (89% predicted), forced vital capacity (FVC) of 2.12 L (103% predicted), total lung capacity (TLC) of 96% predicted, and diffusing capacity of the lungs for carbon monoxide (DLCO) of 62% predicted. Her Eastern Cooperative Oncology Group (ECOG) performance status was 0.

Following multidisciplinary discussion, surgical resection was selected based on the patient's operability, pulmonary reserve, prior treatment history, and overall clinical circumstances.

Following multidisciplinary discussion, surgical resection was selected based on the patient's operability, pulmonary reserve, prior treatment history, and overall clinical circumstances.

At Week 9, she underwent robotic-assisted superior segmentectomy of the left lower lobe (S6) with additional margin resection and systematic mediastinal and hilar lymph node dissection. Final pathology demonstrated a 2.2 cm well-differentiated adenosquamous carcinoma composed of adenocarcinoma and squamous cell carcinoma components (Figures 9, 10), confined to the lung parenchyma. The initial parenchymal margin was focally 1 mm from the tumor, and the separately submitted final additional margin was negative. All sampled lymph nodes were negative for malignancy. PD-L1 immunohistochemistry using 22C3 demonstrated weak expression with a tumor proportion score of 2%.

**Figure 9:**
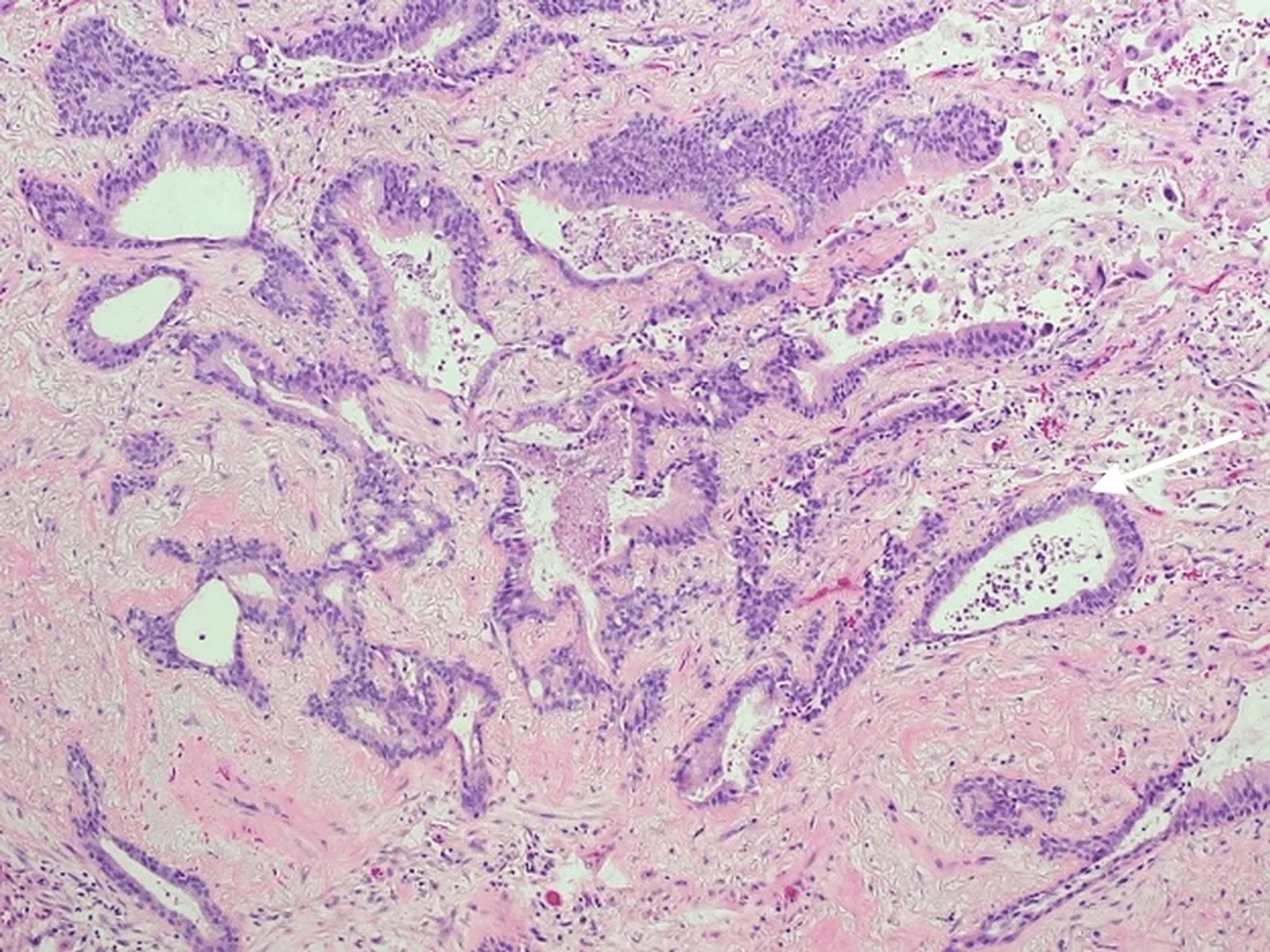
Surgical pathology demonstrating adenocarcinoma component of the resected lesion. H&E stain (×100) from the left lower lobe superior segmentectomy specimen demonstrating well-differentiated adenocarcinoma. H&E, hematoxylin and eosin.

**Figure 10:**
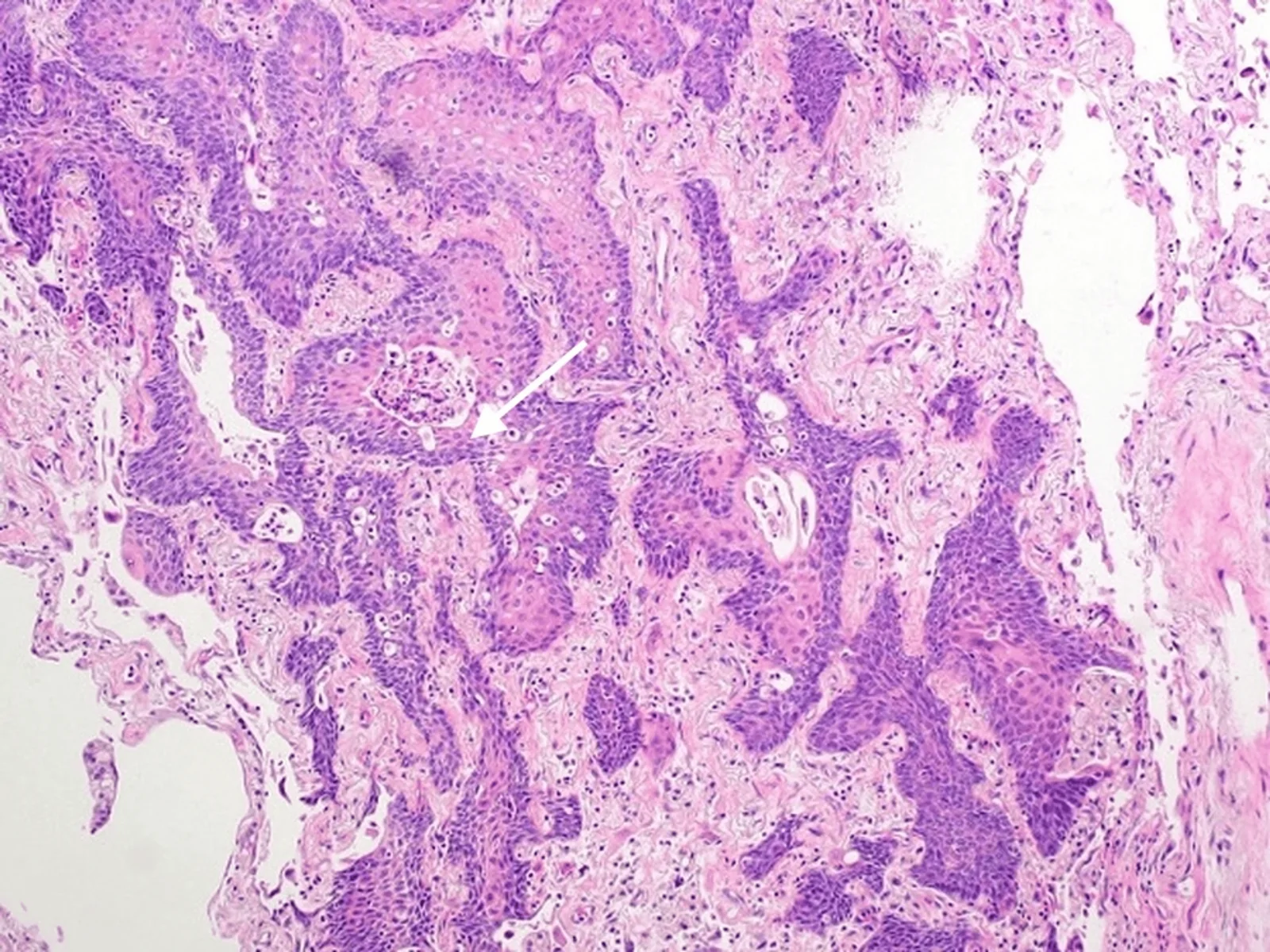
Surgical pathology demonstrating squamous component of adenosquamous carcinoma. H&E stain (×100) from the left lower lobe superior segmentectomy specimen demonstrating irregular sheets of keratinizing squamous cell carcinoma, confirming adenosquamous carcinoma. H&E, hematoxylin and eosin.

Postoperatively, she recovered well with minimal pain and early ambulation and was discharged home on Postoperative Day 2. At approximately seven months after resection, surveillance imaging demonstrated no new pulmonary nodules or masses and no radiographic evidence of recurrent thoracic malignancy. A summary of the patient’s oncologic history, diagnostic evaluation, and management is provided in Table 1.

**Table 1:** Clinical timeline of the patient’s oncologic history, diagnostic evaluation, and management. The timeline summarizes the patient’s initial diagnosis and treatment of myxofibrosarcoma, subsequent local recurrence and re-irradiation, development and diagnostic evaluation of the left lower lobe pulmonary lesion, and definitive surgical management. Time points are presented relative to the initial sarcoma diagnosis and subsequent pulmonary lesion detection. SUVmax, maximum standardized uptake value; rEBUS, radial endobronchial ultrasound; CBCT, cone-beam computed tomography.

Time point	Clinical event
Year 0	Initial 4 cm myxofibrosarcoma excised with positive margins
Year 0	Radical re-excision demonstrated residual tumor ≤1 cm with negative but close margins
Year 0	Adjuvant radiotherapy, 60 Gy in 30 fractions
Year 10	High-grade local myxofibrosarcoma recurrence
Year 10	Radical re-excision of 4.2 cm recurrent tumor with negative but close margins
Year 10	Whole-body PET/CT showed no distant metastatic disease
Year 10	Electron re-irradiation, 50 Gy in 25 fractions plus 10 Gy boost in five fractions
Day 0	Surveillance CT demonstrated enlarging spiculated/fibrotic left lower lobe abnormality
Shortly after Day 0	PET/CT demonstrated 1.9 cm spiculated LLL nodule, SUVmax 2.9
Week 5	Robotic bronchoscopy with rEBUS, CBCT, augmented fluoroscopy, and cryobiopsy
Week 8	Preoperative pulmonary function testing
Week 9	Robotic S6 superior segmentectomy with lymph node dissection
Approximately 7 months after surgery	Surveillance imaging showed no new pulmonary nodules or masses

## Discussion

Management of a new pulmonary lesion in a patient with prior chest-wall irradiation is particularly challenging because post-treatment parenchymal changes can closely mimic scarring or pulmonary malignancy. In this setting, PET imaging can help identify metabolically active areas that warrant further evaluation, although FDG uptake is not specific for malignancy.

In our case, PET/CT demonstrated a 1.9 cm spiculated pulmonary nodule with an SUVmax of 2.9. The combination of interval progression, suspicious morphology, and focal FDG uptake increased concern for malignancy. Radiation exposure to adjacent lung tissue may also result in chronic inflammation, fibrosis, traction bronchiectasis, and architectural distortion, which can further complicate radiographic interpretation [1-3,7].

Several mechanisms have been proposed to contribute to radiation-associated carcinogenesis, including persistent endothelial injury, oxidative stress, genomic instability, and impaired DNA repair [1-8]. However, these mechanisms remain incompletely established. Although population-based studies have demonstrated an increased risk of second primary malignancies following selected thoracic radiation exposures, causal attribution in an individual patient remains difficult.

In this patient, the pulmonary malignancy developed approximately 11 years after the initial chest-wall radiation course and was histologically distinct from the original myxofibrosarcoma. These features raised consideration of a radiation-associated second primary malignancy. However, the available dosimetric information requires caution in making this association.

Review of the available electron boost plan demonstrated a mean dose of 0.889 Gy and a maximum dose of 10.625 Gy to the contoured lungs excluding the gross target volume. These values represent the boost plan only and do not reflect cumulative dose from both radiation courses. In addition, the later pulmonary tumor was not prospectively contoured on the radiation plan, and the exact cumulative dose at the lesion site could not be determined. Therefore, while the lesion developed adjacent to a previously irradiated posterior thoracic region, a causal relationship between radiation exposure and tumor development cannot be established from this case.

The primary procedural challenge in this case was accurate localization of the intended target in the setting of surrounding post-treatment architectural distortion. Cone-beam CT, augmented fluoroscopy, robotic bronchoscopy, and radial EBUS were used together to localize the posterior lesion and confirm tool-in-lesion positioning prior to sampling.

The augmented fluoroscopic pathway was particularly useful for maintaining visualization of the target during respiratory motion. The green circular overlays shown in Figure 5 represent the targeting pathway used to maintain lesion localization throughout inspiration and expiration, reducing the risk of losing focus of the target during navigation.

Posterior lung lesions may also be affected by dependent atelectasis and CT-to-body divergence. Physiologic principles described in the prone-positioning literature support the use of prone positioning when targeting selected posterior lower-lobe lesions [9]. In this patient, prone positioning was used to reduce dependent posterior atelectasis and facilitate lesion localization.

This multimodal approach successfully resulted in diagnostic tissue acquisition. However, this case does not establish that robotic bronchoscopy, rEBUS, CBCT, or augmented fluoroscopy is superior to other diagnostic approaches. Similarly, although targeted sampling may have reduced sampling of adjacent fibrotic tissue, this potential benefit was not directly measured.

Once malignancy was confirmed, definitive management required individualized multidisciplinary evaluation. The patient had excellent performance status, preserved spirometry and lung volumes, and moderately reduced diffusion capacity. Surgical resection was selected based on these factors together with her prior treatment history and overall clinical circumstances.

Recent studies, including randomized trials, support sublobar resection as an appropriate treatment option in selected patients with small peripheral early-stage non-small cell lung cancer (NSCLC) [10-13]. In our patient, minimally invasive superior segmentectomy achieved complete resection with a negative final margin and negative sampled lymph nodes while preserving uninvolved lung parenchyma. However, this case does not establish superiority over lobectomy, SBRT, or other treatment strategies, and long-term oncologic or functional benefit cannot be determined from the available follow-up.

The final diagnosis of adenosquamous carcinoma is clinically relevant because this subtype has been described as an aggressive histologic subtype with a substantial risk of postoperative recurrence in published series [14,15]. This is a literature-based observation and should not be interpreted as establishing the prognosis of this individual patient.

Post-treatment surveillance is also important in patients with complex thoracic treatment histories because fibrosis and architectural distortion may complicate recognition of recurrent or new disease. In this patient, surveillance imaging approximately seven months after surgery demonstrated no new pulmonary nodules or masses. This follow-up remains limited, and no conclusions regarding long-term recurrence or survival can be made.

Overall, this case highlights the importance of careful correlation of prior treatment history, serial imaging, PET findings, and procedural planning when evaluating suspicious pulmonary lesions in post-treatment thoracic anatomy. Multimodal image-guided bronchoscopy successfully facilitated lesion localization and tissue diagnosis in this patient, although larger studies are needed to determine its comparative diagnostic value.

## Conclusions

Radiation-associated lung malignancy remains an uncommon but clinically important consideration following thoracic and chest-wall radiotherapy. Chronic post-treatment fibrosis and architectural distortion may complicate the evaluation of new pulmonary lesions and make accurate lesion localization more challenging.

This case illustrates the successful use of RAB, radial EBUS, CBCT, and augmented fluoroscopy to localize and obtain diagnostic tissue from a posterior left lower lobe lesion in complex post-treatment thoracic anatomy. Careful review of the patient's clinical history, serial imaging, and PET findings contributed to target selection; however, the relative importance of lesion-selection strategy versus the individual technologies cannot be determined from a single case.

Although the temporal and anatomic relationship to prior chest-wall radiotherapy raised consideration of a radiation-associated second primary malignancy, available dosimetric information does not permit definitive causal attribution. Similarly, the successful use of multimodal bronchoscopy in this patient does not establish improved diagnostic accuracy or superiority over alternative approaches.

Following individualized multidisciplinary evaluation, lung-sparing surgical resection achieved complete resection with a negative final margin and negative sampled lymph nodes. Short-term surveillance demonstrated no radiographic evidence of recurrent thoracic malignancy, although long-term oncologic and functional outcomes remain unknown.

This case emphasizes the importance of careful lesion localization and targeted tissue acquisition when evaluating suspicious pulmonary lesions in patients with complex post-treatment thoracic anatomy.
